# Weight-based polymyxin B dosing for mortality and nephrotoxicity: an exploratory dose–response study

**DOI:** 10.1093/jac/dkag322

**Published:** 2026-09-18

**Authors:** Sung-Ching Pan, Ming-Tao Tsai, Jann-Tay Wang, Wang-Huei Sheng, Yu-Chung Chuang

**Affiliations:** Department of Internal Medicine, National Taiwan University Hospital, Taipei, Taiwan; Department of Medicine, National Taiwan University Cancer Center, Taipei, Taiwan; Department of Internal Medicine, National Taiwan University Hospital, Taipei, Taiwan; Department of Internal Medicine, National Taiwan University Hospital, Taipei, Taiwan; Department of Internal Medicine, National Taiwan University Hospital, Taipei, Taiwan

## Abstract

**Objectives:**

The optimal weight-based maintenance dose of polymyxin B is not established. We evaluated dose–response relationships for 28-day mortality and nephrotoxicity and explored a dose threshold.

**Methods:**

In this single-centre retrospective cohort, hospitalized adults received a first intravenous polymyxin B course (June 2025 to February 2026); the maintenance dose (mg/kg/day) was the exposure. Main outcomes were 28-day mortality and nephrotoxicity, defined by a creatinine-clearance criterion (≥50% fall, or  ≥ 25% with pre-existing dysfunction); renal injury was also graded by the risk, injury, failure, loss and end-stage kidney disease (RIFLE) classification. We used clinically selected dose categories (2.5, 2.75 and 3.0 mg/kg/day), exploratory classification and regression tree (CART) analysis, multivariable logistic regression and a desirability of outcome ranking.

**Results:**

The mortality cohort comprised 208 patients (28-day mortality 41.3%) and the nephrotoxicity cohort 163 (nephrotoxicity 49.7%). Mortality declined across dose categories in univariable analysis (trend *P* = 0.05) but was not independently associated with dose after adjustment. Nephrotoxicity increased with dose, reaching 73% at  ≥3.0 mg/kg/day; CART identified a threshold at 2.74 mg/kg/day, above which the RIFLE grade was worse (62.3% of comparisons; *P* = 0.006). Multivariable analysis confirmed this (adjusted odds ratio 2.60, 95% confidence interval 1.25–5.43). The desirability of outcome ranking did not differ across dose groups.

**Conclusions:**

A higher polymyxin B maintenance dose was associated with increased nephrotoxicity, particularly above approximately 2.75 mg/kg/day. The mortality estimates were imprecise and did not exclude a clinically important effect. This single-centre threshold is hypothesis-generating and requires validation before it could inform a clinical dose ceiling.

## Introduction

Carbapenem-resistant Gram-negative bacteria, in particular carbapenem-resistant *Acinetobacter baumannii*, *Pseudomonas aeruginosa* and Enterobacterales such as *Klebsiella pneumoniae*, are among the highest-priority antimicrobial-resistance threats and a frequent cause of difficult-to-treat infection in critically ill patients,^1,2^ and a leading contributor to the global mortality attributable to bacterial antimicrobial resistance.^3^ Although newer agents such as ceftazidime-avibactam and cefiderocol are increasingly used, their availability and pathogen coverage vary, and the polymyxins remain an important reserve option, particularly within combination regimens for carbapenem-resistant *A. baumannii*.^4,5^ Of the two polymyxins in clinical use, polymyxin B is preferred over colistin because it is less nephrotoxic,^6–8^ although both cause dose-dependent nephrotoxicity.^9–11^

The 2019 international consensus recommends a polymyxin B maintenance dose of 1.25–1.5 mg/kg every 12 h (2.5–3.0 mg/kg/day), derived from pharmacokinetic simulation to reach an exposure adequate for efficacy and capped at 3 mg/kg/day because safety data above that level were lacking; the consensus itself notes that exposure-toxicity data for polymyxin B are scarce, the therapeutic window is narrow and dose-optimized studies are needed.^6^ Whether the recommended range is optimal has not been established by a rigorous statistical method. A systematic review of studies graded by the risk, injury, failure, loss and end-stage kidney disease (RIFLE) criteria found a higher daily polymyxin dose to be an independent risk factor for nephrotoxicity, but treated dose as a continuous predictor without deriving a threshold.^10^ An exposure-based study derived an area-under-the-curve threshold for nephrotoxicity,^12^ but this describes drug exposure rather than the administered dose and requires therapeutic drug monitoring, which is not routinely available. Weight-based dose evidence is sparse: the single dose-mortality study used a cohort-median cut in largely renal-impaired patients^13^ and high-dose cohorts report frequent kidney injury without deriving a threshold.^14,15^

Mortality in critically ill patients is multifactorial and cannot be attributed to any single exposure. We nevertheless pre-specified 28-day all-cause mortality as the primary outcome because it is objective, ascertained completely, free of the attribution bias that affects infection-related mortality and commonly used in treatment studies of carbapenem-resistant Gram-negative infection,^16^ and because it captures the potential survival benefit against which dose-related harm must be weighed. Nephrotoxicity is the biologically plausible dose-dependent harm, and the two outcomes were therefore assessed together.

We therefore conducted an exploratory dose–response analysis of polymyxin B for 28-day mortality and for nephrotoxicity defined by a creatinine-clearance criterion corroborated by the RIFLE classification.^17,18^ We examined whether a clinically relevant weight-based dose threshold could be identified.

## Patients and methods

### Ethics

The Research Ethics Committee of National Taiwan University Hospital approved the study (approval number 202603145RINE) and waived the requirement for informed consent owing to the retrospective design. The study was conducted in accordance with the Declaration of Helsinki and national and institutional standards.

### Study design and setting

This single-centre, retrospective cohort study was conducted at National Taiwan University Hospital, a tertiary referral centre in Taipei. Eligible patients received intravenous polymyxin B between 1 June 2025 and 28 February 2026 soon after the drug became available in Taiwan.

### Study population

We included hospitalized adults aged 18 years or older and analysed only the first polymyxin B course per patient. A course was consecutive daily administration, and a gap of more than 1 day defined a separate course. All patients were required to have received at least two consecutive days of polymyxin B; those given only a loading dose or a single day of therapy were excluded. This minimum applied to both the mortality and the nephrotoxicity cohorts.

### Data collection and definitions

Clinical data and laboratory values were abstracted from the electronic medical record. Day 0 was defined as the polymyxin B start date. The quick Sequential Organ Failure Assessment (qSOFA) score^19,20^ and the Pitt bacteraemia score^21^ were taken at Day 0, and comorbidity burden was summarized by the Charlson comorbidity index.^22^ The infection focus and causative pathogen were assigned by the treating physician. A pathogen was carbapenem-resistant if resistant to ertapenem, meropenem or imipenem (Enterobacterales) or to meropenem or imipenem (non-fermenters). Polymyxin susceptibility was inferred from the Vitek 2 colistin minimum inhibitory concentration, with a value of above 2 mg/L interpreted as resistant (Clinical and Laboratory Standards Institute criteria)^23^; reference polymyxin B minimum inhibitory concentration testing was not performed.

The institutionally recommended maintenance dose was 1.25–1.5 mg/kg every 12 h, preceded by a loading dose. The maintenance dose (mg/kg/day on total body weight) was taken from the first administered maintenance regimen and analysed as the baseline exposure; the loading dose was the single, higher first administration preceding the maintenance regimen. Concomitant antibacterial and nephrotoxic agents, including inhaled colistin, were recorded.

### Outcomes

The primary outcome was 28-day all-cause mortality and the principal safety outcome was nephrotoxicity; both were analysed in parallel throughout. Twenty-eight days was chosen because it is the interval conventionally used in studies of acute Gram-negative infection and spans the treatment course and its immediate aftermath.^16^

The nephrotoxicity-evaluable cohort excluded patients on chronic renal replacement therapy and those without a baseline creatinine. The baseline creatinine was the value measured on the day of the first polymyxin B dose where available, or otherwise the nearest value within 1 day before or after. Nephrotoxicity was assessed during the course by a creatinine-clearance criterion tiered by baseline renal function (Cockcroft-Gault^24^ clearance ≥80 versus <80 mL/min): a fall of at least 50% in calculated creatinine clearance with normal baseline function (the RIFLE Injury threshold)^17^ and a more sensitive fall of at least 25% with pre-existing renal dysfunction (the RIFLE Risk threshold). As a separate, broader measure, renal injury was also graded by the full RIFLE classification (risk through end-stage), which captures renal outcomes beyond the treatment course, including the loss and end-stage stages defined by renal replacement therapy at 4 weeks and 3 months.

Because a higher dose could plausibly reduce mortality while increasing nephrotoxicity, we combined the two outcomes for each nephrotoxicity-evaluable patient in a desirability of outcome ranking (DOOR),^25^ which places every patient on a single ordered clinical scale: Rank 1, alive without nephrotoxicity; Rank 2, alive with nephrotoxicity; Rank 3, death. A DOOR probability near 50% indicates that a patient chosen at random from one dose group is no more likely to have a better outcome than a patient chosen at random from the comparator group.

### Statistical analysis

Continuous variables are summarized as median (interquartile range) and categorical variables as number (percentage), compared with the Mann–Whitney *U*, chi-square or Fisher exact test. Logistic regression was the primary method for each binary outcome: candidate variables with *P* < 0.15 on univariable analysis entered backward elimination by the Akaike information criterion, and the final model retained variables with *P* < 0.05. For the mortality model, treatment duration and cumulative dose were ineligible because of immortal-time bias and creatinine and creatinine clearance because they are unreliable during renal replacement therapy.

Baseline characteristics were compared using observed values, without imputation. Models were fitted using complete cases, and multiple imputation by chained equations was used as a sensitivity analysis.^26^ Because qSOFA does not grade organ failure comprehensively,^20^ the models for both outcomes were also repeated as sensitivity analyses with disease-severity indicators (the Pitt bacteraemia score, its mechanical-ventilation and hypotension components and intensive care unit status at initiation) added as further covariates, each in a separate model.

Time-to-event outcomes used the same variables: Kaplan–Meier estimates with the log-rank test and Cox regression for mortality and a Fine–Gray subdistribution hazard model^27^ with death as a competing risk, compared by Gray’s test, for nephrotoxicity. The dose–response relationship was examined with a generalized additive model (penalized spline); with the dose categorized into three and four pre-specified ordered levels at 2.5, 2.75 and 3.0 mg/kg/day (the low, middle and high of the consensus range; left-closed, so each cut point fell in the higher category; trend tested by a linear term for category rank); and with a single-split classification and regression tree (CART) whose cutpoint was summarized as the median of 2000 bootstrap resamples (fixed seed) with its 95% confidence interval. Each binary outcome was dichotomized at its CART-derived cutpoint or, where CART identified no cutpoint, at the pre-specified lower bound of the consensus range (2.5 mg/kg/day), contrasting under-dosing with dosing within the recommended range. The ordinal RIFLE grade and DOOR were compared across dose groups by the Mann–Whitney relative effect with bootstrap confidence intervals, and RIFLE was additionally modelled by ordinal proportional-odds regression. Analyses used R 4.5.1, with two-sided *P* < 0.05 considered significant.

## Results

### Baseline characteristics

**Figure 1.**
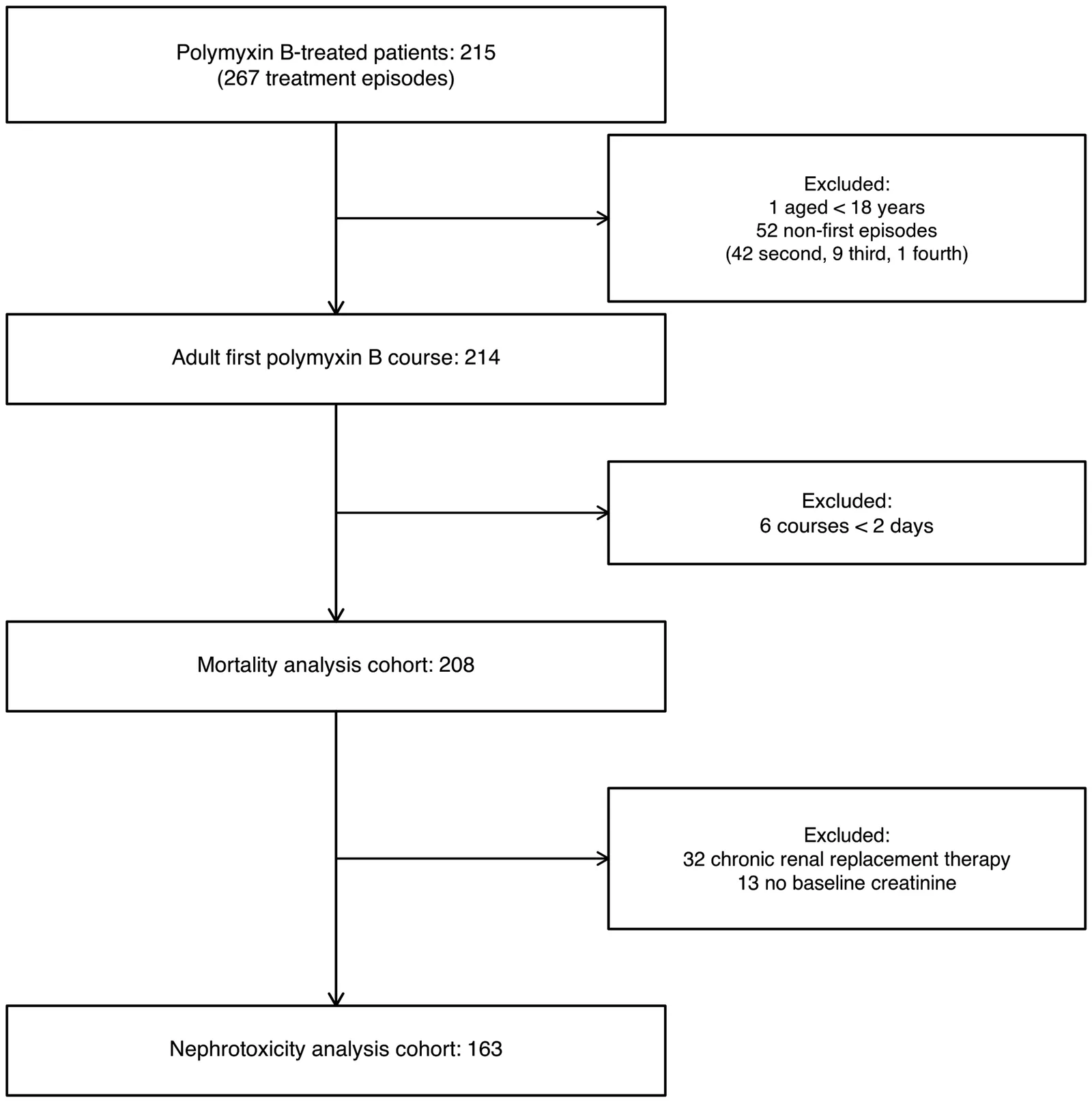
Flow of patients through the study and derivation of the mortality and nephrotoxicity analysis cohorts.

During the study period, 215 patients received a first polymyxin B course; after excluding one patient younger than 18 years, 214 adults formed the study population, of whom 208 had received polymyxin B on at least two consecutive days (mortality cohort) and 163 were nephrotoxicity-evaluable (Figure 1). The cohort was critically ill; baseline characteristics are summarized in Table 1. Pneumonia was the most common infection (146 patients, 70.2%), followed by bloodstream infection (30, 14.4%).

**Table 1.** Baseline characteristics of the mortality cohort, by 28-day mortality

Variable^a^	Total (*n* = 208)	Survivors (*n* = 122)	Non-survivors (*n* = 86)	*P*-value
Age, years	69.0 [59.0–78.2]	68.5 [58.2–77.0]	71.0 [60.2–79.8]	0.16
Male sex	135 (64.9%)	80 (65.6%)	55 (64.0%)	0.93
Body weight, kg	57.8 [50.7–67.0]	57.7 [50.7–67.0]	58.5 [50.7–66.3]	0.93
Body mass index, kg/m^2^	21.9 [19.2–24.6]	21.9 [19.0–24.4]	21.9 [19.6–24.8]	0.60
Charlson comorbidity index	6.0 [4.0–8.0]	5.0 [4.0–7.8]	6.0 [4.0–8.0]	0.14
Diabetes mellitus	65 (31.2%)	41 (33.6%)	24 (27.9%)	0.47
Liver cirrhosis	42 (20.2%)	23 (18.9%)	19 (22.1%)	0.69
Chronic kidney disease	32 (15.4%)	16 (13.1%)	16 (18.6%)	0.38
Malignancy	129 (62.0%)	72 (59.0%)	57 (66.3%)	0.36
Immunosuppressant use	91 (43.8%)	52 (42.6%)	39 (45.3%)	0.80
Systemic corticosteroid use	69 (33.2%)	38 (31.1%)	31 (36.0%)	0.56
White blood cell count, ×10^3^/µL	9.9 [6.0–16.9]	10.1 [6.8–15.0]	9.9 [4.2–18.0]	0.96
Platelet count, ×10^3^/µL	132 [47–229]	166 [79–271]	82 [38–168]	<0.001
C-reactive protein, mg/dL	9.9 [6.0–15.0]	7.8 [4.5–12.1]	12.2 [8.9–17.2]	<0.001
Serum albumin, g/dL	2.9 [2.6–3.3]	3.1 [2.7–3.4]	2.8 [2.5–3.1]	<0.001
Baseline serum creatinine, mg/dL	0.77 [0.46–1.63]	0.67 [0.45–1.25]	0.97 [0.58–1.94]	0.007
Creatinine clearance^b^, mL/min	71.9 [33.8–110.3]	79.9 [45.2–128.4]	49.5 [26.9–93.1]	0.004
Carbapenem-resistant pathogen identified	161 (77.4%)	97 (79.5%)	64 (74.4%)	0.49
Empirical polymyxin B therapy^c^	164 (78.8%)	93 (76.2%)	71 (82.6%)	0.35
Pneumonia	146 (70.2%)	86 (70.5%)	60 (69.8%)	>0.99
Bloodstream infection	30 (14.4%)	20 (16.4%)	10 (11.6%)	0.45
qSOFA score	2.0 [1.0–3.0]	2.0 [1.0–3.0]	3.0 [2.0–3.0]	<0.001
Pitt bacteraemia score	4.0 [2.0–6.0]	4.0 [2.0–5.8]	4.0 [2.0–6.0]	0.22
Intensive care unit at polymyxin B initiation	85 (40.9%)	45 (36.9%)	40 (46.5%)	0.21
Loading dose	205 (98.6%)	120 (98.4%)	85 (98.8%)	>0.99
Loading dose, mg/kg	2.33 [2.16–2.46]	2.35 [2.17–2.48]	2.31 [2.15–2.43]	0.04
Maintenance dose, mg/kg/day	2.78 [2.55–2.93]	2.80 [2.58–2.95]	2.75 [2.47–2.87]	0.09
Polymyxin B duration, days	8 [4–12]	9 [5–14]	6 [4–10]	<0.001
Cumulative dose, mg/kg	20.0 [11.9–31.6]	24.0 [15.0–34.8]	15.1 [10.5–25.8]	<0.001
Concomitant antibacterial agent use^d^	200 (96.2%)	118 (96.7%)	82 (95.3%)	0.72
Inhaled colistin	107 (51.4%)	62 (50.8%)	45 (52.3%)	0.94
Aminoglycoside	14 (6.7%)	9 (7.4%)	5 (5.8%)	0.87
Glycopeptide	56 (26.9%)	33 (27.0%)	23 (26.7%)	>0.99
Trimethoprim-sulfamethoxazole	47 (22.6%)	27 (22.1%)	20 (23.3%)	0.98
Amphotericin B	11 (5.3%)	4 (3.3%)	7 (8.1%)	0.21

Missing laboratory values: C-reactive protein 33 (15.9%), baseline serum creatinine and creatinine clearance 26 (12.5%), serum albumin 10 (4.8%), white blood cell count 4 (1.9%) and platelet count 4 (1.9%); all other variables were complete. Comparisons used observed values only. Thirteen of the 26 patients without a baseline serum creatinine were receiving chronic renal replacement therapy and were excluded from the nephrotoxicity cohort on that basis.

^a^Data are represented as *n* (%) or median (interquartile range).

^b^Creatinine clearance estimated by the Cockcroft-Gault equation.

^c^Empirical polymyxin B therapy, started before the susceptibility result.

^d^Concomitant antibacterial agent use, any agent overlapping the polymyxin B course.

Polymyxin B was started after a carbapenem-resistant organism was identified in 44 patients and empirically in 164, of whom 117 were later confirmed; in total, 161 patients (77.4%) were treated for a carbapenem-resistant pathogen (73 *A. baumannii*, 37 *P. aeruginosa*, 33 *K. pneumoniae* and 18 other Enterobacterales).

### Factors associated with 28-day mortality

Eighty-six of the 208 patients (41.3%) died within 28 days; univariable comparisons are shown in Table 1. In multivariable logistic regression, older age [adjusted odds ratio (aOR) 1.03, 95% confidence interval (CI) 1.01–1.06; *P* = 0.02], a higher qSOFA score (aOR 1.76, 95% CI 1.17–2.65; *P* = 0.007), a higher C-reactive protein (aOR 1.06, 95% CI 1.01–1.11; *P* = 0.02), a lower serum albumin (aOR 0.51, 95% CI 0.26–0.99; *P* = 0.047) and a lower platelet count (aOR 0.95 per 10^4^/µL, 95% CI 0.92–0.98; *P* = 0.003) were independently associated with mortality (Table 2). The maintenance dose was not associated with mortality as a continuous variable (aOR 0.41, 95% CI 0.12–1.37; *P* = 0.15). Because no data-driven cutpoint was identified for mortality, the dose was dichotomized at the pre-specified lower bound of the consensus range (2.5 mg/kg/day), which was likewise not associated with mortality (aOR 0.55, 95% CI 0.23–1.30; *P* = 0.17); survival did not differ across this cut (log-rank *P* = 0.12), and the dose remained non-significant in the Cox model (Figure S1a, available as Supplementary data at *JAC* Online, and Table S1). In a sensitivity analysis using multiple imputation for the incomplete laboratory values, the final model was essentially unchanged (Table S2) and the maintenance dose remained non-significant, whether continuous (aOR 0.45, 95% CI 0.15–1.34; *P* = 0.15) or dichotomized at 2.5 mg/kg/day (aOR 0.53, 95% CI 0.24–1.14; *P* = 0.11). Adding the disease-severity indicators left these estimates essentially unchanged, whether the dose was continuous (aOR 0.39–0.43) or dichotomized at 2.5 mg/kg/day (aOR 0.54–0.58); the dose remained not significantly associated with mortality (Table S3).

**Table 2.** Multivariable logistic regression for 28-day mortality

Predictor	Model 1^a^		Model 2^b^		Model 3^c^	
	aOR (95% CI)	*P*-value	aOR (95% CI)	*P*-value	aOR (95% CI)	*P-*value
Age, years	1.03 (1.01–1.06)	0.02	1.03 (1.00–1.06)	0.02	1.03 (1.00–1.06)	0.02
qSOFA, point	1.76 (1.17–2.65)	0.007	1.75 (1.16–2.64)	0.008	1.82 (1.19–2.76)	0.005
C-reactive protein, mg/dL	1.06 (1.01–1.11)	0.02	1.06 (1.01–1.11)	0.02	1.06 (1.01–1.11)	0.02
Serum albumin, g/dL	0.51 (0.26–0.99)	0.047	0.56 (0.29–1.10)	0.09	0.55 (0.28–1.07)	0.08
Platelet count, 10^4^/µL	0.95 (0.92–0.98)	0.003	0.95 (0.92–0.98)	0.002	0.95 (0.92–0.98)	0.003
Maintenance dose, mg/kg/day	–	–	0.41 (0.12–1.37)	0.15	–	–
Dose ≥2.5 mg/kg/day	–	–	–	–	0.55 (0.23–1.30)	0.17

The models were fitted on the 169 of 208 patients (81.2%) with complete data for the retained covariates.

Abbreviations: aOR, adjusted odds ratio; qSOFA, quick Sequential Organ Failure Assessment.

^a^Final multivariable model; variables retained after backward elimination (Akaike information criterion) at *P* < 0.05.

^b^With the continuous polymyxin B maintenance dose forced in.

^c^With the dichotomized polymyxin B dose (≥2.5 mg/kg/day) forced in.

### Factors associated with nephrotoxicity

Eighty-one of the 163 patients (49.7%) developed nephrotoxicity during treatment; univariable comparisons are shown in Table 3. In multivariable logistic regression, older age (aOR 1.04, 95% CI 1.02–1.07; *P* = 0.002), longer treatment duration (aOR 1.16 per day, 95% CI 1.07–1.25; *P* < 0.001) and a higher continuous maintenance dose (aOR 3.94, 95% CI 1.17–13.27; *P* = 0.03) were independently associated with nephrotoxicity, whereas corticosteroid use was inversely associated (aOR 0.37, 95% CI 0.16–0.82; *P* = 0.01) (Table 4). The Fine–Gray and ordinal RIFLE models gave consistent dose estimates (Tables S4 and S5).

**Table 3.** Baseline characteristics of the nephrotoxicity-evaluable cohort, by nephrotoxicity

Variable^a^	Total (*n* = 163)	No nephrotoxicity (*n* = 82)	Nephrotoxicity (*n* = 81)	*P*-value
Age, years	69.0 [59.5–79.0]	66.0 [54.0–76.0]	72.0 [65.0–79.0]	0.006
Male sex	106 (65.0%)	51 (62.2%)	55 (67.9%)	0.55
Body weight, kg	57.7 [50.5–66.0]	58.5 [50.3–66.6]	56.8 [50.6–66.0]	0.98
Body mass index, kg/m^2^	21.6 [18.9–24.2]	21.7 [19.0–24.7]	21.6 [18.8–23.9]	0.79
Charlson comorbidity index	6.0 [4.0–8.0]	5.0 [4.0–7.0]	6.0 [4.0–8.0]	0.045
Diabetes mellitus	52 (31.9%)	23 (28.0%)	29 (35.8%)	0.37
Liver cirrhosis	30 (18.4%)	14 (17.1%)	16 (19.8%)	0.81
Chronic kidney disease	12 (7.4%)	3 (3.7%)	9 (11.1%)	0.13
Malignancy	108 (66.3%)	53 (64.6%)	55 (67.9%)	0.78
Immunosuppressant use	67 (41.1%)	40 (48.8%)	27 (33.3%)	0.07
Systemic corticosteroid use	47 (28.8%)	31 (37.8%)	16 (19.8%)	0.02
White blood cell count, ×10^3^/µL	9.0 [5.8–17.0]	9.0 [4.3–17.5]	9.0 [6.4–14.9]	0.46
Platelet count, ×10^3^/µL	148 [50–238]	137 [36–243]	160 [81–235]	0.22
C-reactive protein, mg/dL	9.3 [6.0–15.1]	9.5 [6.3–18.1]	9.0 [5.4–13.8]	0.34
Serum albumin, g/dL	2.9 [2.6–3.3]	3.0 [2.6–3.4]	2.9 [2.6–3.3]	0.63
Baseline serum creatinine, mg/dL	0.69 [0.45–1.38]	0.65 [0.41–1.89]	0.77 [0.56–1.16]	0.50
Creatinine clearance^b^, mL/min	74.1 [37.6–116.7]	86.6 [32.4–132.2]	69.3 [43.9–93.7]	0.25
Carbapenem-resistant pathogen identified	123 (75.5%)	57 (69.5%)	66 (81.5%)	0.11
Empirical Polymyxin B therapy^c^	127 (77.9%)	64 (78.0%)	63 (77.8%)	>0.99
Pneumonia	116 (71.2%)	58 (70.7%)	58 (71.6%)	>0.99
Bloodstream infection	24 (14.7%)	13 (15.9%)	11 (13.6%)	0.85
qSOFA score	2.0 [1.0–3.0]	2.0 [1.2–3.0]	2.0 [1.0–3.0]	0.13
Pitt bacteraemia score	3.0 [2.0–5.5]	4.0 [2.0–5.0]	3.0 [1.0–6.0]	0.20
Intensive care unit at polymyxin B initiation	61 (37.4%)	32 (39.0%)	29 (35.8%)	0.79
Loading dose	161 (98.8%)	80 (97.6%)	81 (100.0%)	0.50
Loading dose, mg/kg	2.33 [2.17–2.46]	2.32 [2.13–2.46]	2.35 [2.22–2.46]	0.33
Maintenance dose, mg/kg/day	2.80 [2.57–2.95]	2.75 [2.50–2.89]	2.83 [2.70–2.97]	0.009
Polymyxin B duration, days	7 [4–11]	5 [3–10]	9 [6–13]	<0.001
Cumulative dose, mg/kg	19.7 [11.8–31.1]	14.3 [9.3–25.2]	24.8 [16.9–35.6]	<0.001
Concomitant antibacterial agent use^d^	156 (95.7%)	77 (93.9%)	79 (97.5%)	0.44
Inhaled colistin	90 (55.2%)	39 (47.6%)	51 (63.0%)	0.07
Aminoglycoside	8 (4.9%)	4 (4.9%)	4 (4.9%)	1.00
Glycopeptide	37 (22.7%)	19 (23.2%)	18 (22.2%)	>0.99
Trimethoprim-sulfamethoxazole	40 (24.5%)	23 (28.0%)	17 (21.0%)	0.39
Amphotericin B	8 (4.9%)	6 (7.3%)	2 (2.5%)	0.28

Abbreviation: qSOFA, quick Sequential Organ Failure Assessment.

^a^Data are represented as *n* (%) or median (interquartile range).

^b^Creatinine clearance estimated by the Cockcroft-Gault equation.

^c^Empirical polymyxin B therapy, started before the susceptibility result.

^d^Concomitant antibacterial agent use, any agent overlapping the polymyxin B course.

**Table 4.** Multivariable logistic regression for nephrotoxicity

Predictor	Continuous-dose model^a^		Dichotomized ≥2.74 model^a^	
	aOR (95% CI)	*P*-value	aOR (95% CI)	*P*-value
Age, years	1.04 (1.02–1.07)	0.002	1.04 (1.02–1.07)	0.002
Polymyxin B duration, days	1.16 (1.07–1.25)	<0.001	1.16 (1.07–1.25)	<0.001
Systemic corticosteroid use	0.37 (0.16–0.82)	0.01	0.37 (0.17–0.84)	0.02
Maintenance dose, mg/kg/day	3.94 (1.17–13.27)	0.03	–	–
Dose ≥2.74 mg/kg/day	–	–	2.60 (1.25–5.43)	0.01

The models were fitted on all 163 nephrotoxicity-evaluable patients; no covariate in these models had missing data.

Abbreviation: aOR, adjusted odds ratio.

^a^Two models differing only in dose parameterization: continuous maintenance dose and dose dichotomized at ≥2.74 mg/kg/day.

### Dose–response relationships

We examined the dose–response across the consensus range (2.5–3.0 mg/kg/day), using 2.5, 2.75 and 3.0 mg/kg/day as pre-specified reference points, with CART as a data-driven check.

**Figure 2.**
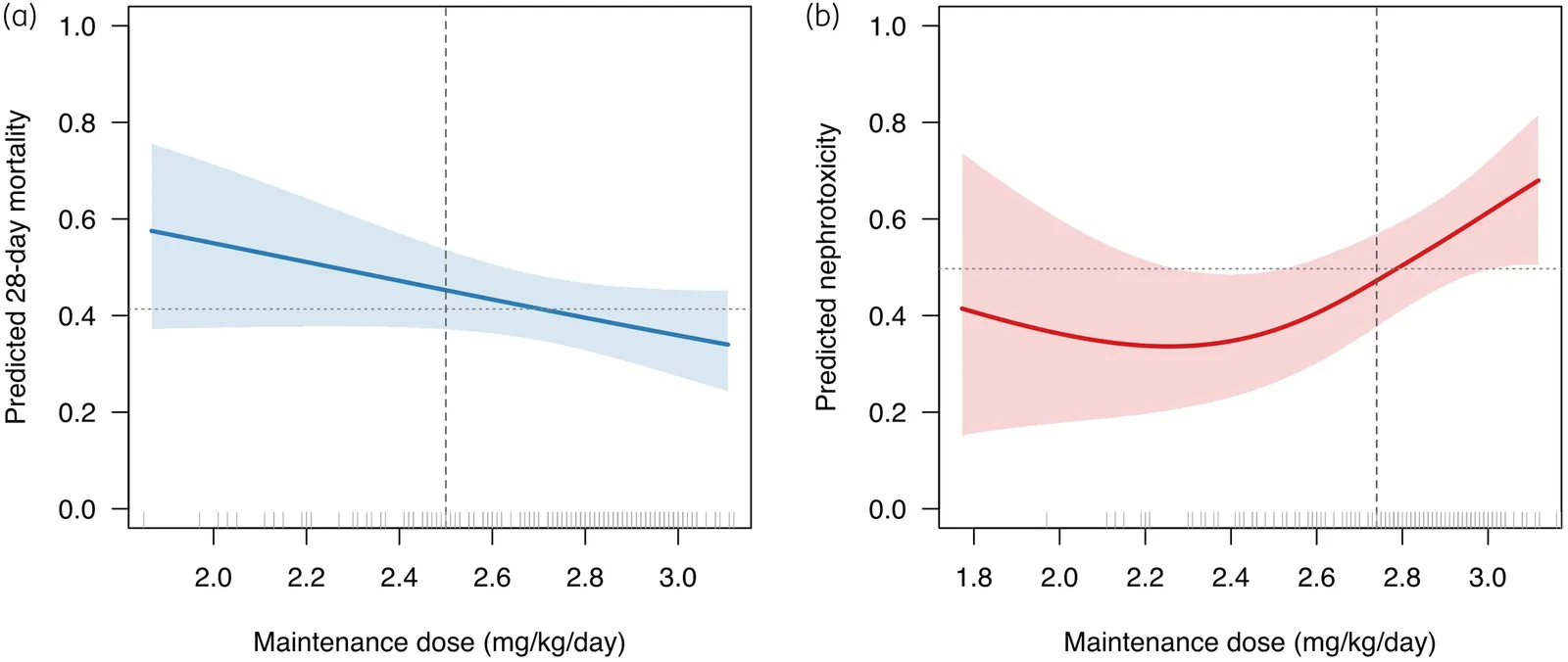
Dose–response relationship between the polymyxin B maintenance dose and outcome. (a) Predicted 28-day mortality and (b) predicted nephrotoxicity as a function of the maintenance dose, modelled by a generalized additive model (penalized spline; fitted curve with 95% confidence interval, shaded). The horizontal dotted line marks the overall observed rate (28-day mortality in (a) and nephrotoxicity in (b)); the vertical dashed line marks the dichotomizing dose (2.5 mg/kg/day in (a) and the data-driven cutpoint of 2.74 mg/kg/day in (b)). The dose–response was significant for nephrotoxicity (edf 2.1, smooth *P* = 0.04) but not mortality (edf 1.00, smooth *P* = 0.10). Abbreviation: edf, effective degrees of freedom.

For mortality, the generalized additive model showed a gradual, non-significant decline (smooth *P* = 0.10; Figure 2a), and 28-day mortality fell from 53% at <2.5 mg/kg/day to 36% at ≥2.75 mg/kg/day (OR 0.50, 95% CI 0.25–1.01; trend *P* = 0.05; Table S6), with no further reduction above 3.0; CART found no cutpoint.

For nephrotoxicity, the additive model showed risk rising above approximately 2.75 mg/kg/day (smooth *P* = 0.04; Figure 2b), and the rate rose from 38% at <2.5 mg/kg/day to 52% at 2.75% to 3.0% and 73% at ≥3.0 mg/kg/day (OR 4.52, 95% CI 1.47–13.92; trend *P* = 0.005; Table S6). CART identified a cutpoint of 2.74 mg/kg/day (bootstrap median; 95% CI 2.54–3.00), essentially the consensus midpoint, and patients were dichotomized at ≥2.74 versus <2.74 mg/kg/day. Above this cutpoint, nephrotoxicity was more frequent in the logistic (aOR 2.60, 95% CI 1.25–5.43; *P* = 0.01), Fine–Gray [subdistribution hazard ratio (sHR) 1.67, 95% CI 1.04–2.67; *P* = 0.03] and ordinal RIFLE (OR 2.12, 95% CI 1.13–3.98; *P* = 0.02) models, with concordant cumulative-incidence (Gray’s *P* = 0.08) and RIFLE-severity (worse 62.3% of the time, *P* = 0.006) results (Figures S1b and S2 and Tables S4 and S5). The maintenance dose was unchanged throughout the course in 151 of the 163 patients (92.6%), reduced in 8 and increased in 4; restricting the model to patients whose dose never changed gave a similar estimate at this cutpoint (aOR 2.53, 95% CI 1.14–5.58; *P* = 0.02). Restricting the baseline creatinine to a value obtained on or before the day of the first dose gave a similar association (153 patients; aOR 2.19, 95% CI 1.03–4.63; *P* = 0.04). Adding the disease-severity indicators left the dose estimate essentially unchanged (aOR 2.58–2.63), and the association with nephrotoxicity remained significant (Table S3).

### DOOR

Because mortality and nephrotoxicity moved in opposite directions as the dose rose, we used the DOOR to test whether any higher-dose group held a net clinical advantage once both outcomes were combined. Among the 163 nephrotoxicity-evaluable patients (Table 5), as the dose increased the proportion of deaths fell (from 50% at <2.5 mg/kg/day to 33% to 38% at ≥2.75 mg/kg/day) but survival with nephrotoxicity rose correspondingly (from 12% to 46%), and survival without nephrotoxicity did not increase. The DOOR analysis did not detect a difference in the distribution of outcome ranks across dose groups: the DOOR probability versus the lowest group was close to 50% throughout (Kruskal–Wallis *P* = 0.70; ≥2.74 versus <2.74, *P* = 0.98). The lower proportion of deaths in the higher-dose groups was accompanied by a higher proportion of survivors with nephrotoxicity, and the overall distribution of outcome ranks did not differ significantly across dose groups.

**Table 5.** Desirability of outcome ranking by polymyxin B maintenance-dose category

Maintenance dose (mg/kg/day)	No. patients	Alive without nephrotoxicity	Alive with nephrotoxicity	Death
<2.5	32	12 (37.5%)	4 (12.5%)	16 (50.0%)
2.5 to <2.75	30	12 (40.0%)	5 (16.7%)	13 (43.3%)
2.75 to <3.0	75	24 (32.0%)	26 (34.7%)	25 (33.3%)
≥3.0	26	4 (15.4%)	12 (46.2%)	10 (38.5%)

Data are number (percentage) of patients within each dose category. The distribution of outcome ranks did not differ significantly across dose groups (Kruskal–Wallis *P* = 0.70).

## Discussion

In this single-centre cohort of critically ill patients receiving polymyxin B, a higher maintenance dose was associated with greater nephrotoxicity, particularly above approximately 2.75 mg/kg/day. This association was consistent across continuous, categorical, time-to-event and RIFLE analyses, whereas maintenance dose was not independently associated with 28-day mortality. Taken together, these findings support an exploratory dose-related renal safety signal above approximately 2.75 mg/kg/day.

Because most patients were critically ill, nephrotoxicity was likely multifactorial and cannot be attributed to dose alone,^9,11^ yet the dose–nephrotoxicity association was observed across logistic regression, Fine–Gray, ordinal RIFLE, the additive model, the categorical gradient and a bootstrapped CART cutpoint. Because the RIFLE loss and end-stage tiers are defined by the duration of renal replacement therapy and are not captured in real time,^17^ we used a creatinine-clearance criterion with a competing-risks model for the time-to-event analysis and graded overall renal injury with the full RIFLE classification; partitioning the RIFLE grade gave the same cutpoint (2.74 mg/kg/day). In the primary model, a dose ≥2.74 mg/kg/day more than doubled the adjusted odds of nephrotoxicity (aOR 2.60, 95% CI 1.25–5.43), and the rate reached 73% at ≥3.0 mg/kg/day. The consistency of the dose association across these analyses supports a renal safety signal near the upper recommended dose range.

The observed renal safety signal can be considered alongside the consensus framework for optimizing the clinical use of the polymyxins.^28^ The 2019 consensus derived 1.25–1.5 mg/kg every 12 h by simulation to reach a steady-state concentration near 2 mg/L for efficacy and capped it at 3 mg/kg/day for lack of safety data rather than from a defined toxicity threshold, noting a narrow window and scarce exposure-toxicity data.^6^ Polymyxin B clearance scales with total body weight and is largely non-renal (0.0276 L/h/kg),^29^ so a weight-based dose gives a predictable exposure without renal adjustment. At that clearance, our cutoff of 2.75 mg/kg/day corresponds to a steady-state area under the curve of approximately 100 mg h/L, compatible with the nephrotoxicity-associated exposure reported by therapeutic drug monitoring in an independent cohort and well above the efficacy threshold of 45.7 mg h/L reported in the same study.^12^ An independent pharmacometric analysis similarly placed the upper, nephrotoxicity-based limit of the polymyxin B exposure window at 100 mg h/L,^30^ a bound the 2019 consensus acknowledged but did not adopt, recommending the lower colistin target in the absence of direct toxicity data.^6^ The clinical dose–response, therapeutic drug-monitoring study and pharmacometric analysis are directionally compatible with increased nephrotoxicity near higher exposures. Because polymyxin B concentrations were not measured in our cohort, the calculated exposure provides pharmacokinetic plausibility rather than validation of a monitoring-free dose-toxicity threshold.

Polymyxin B killing is concentration-dependent, best described by the ratio of the free-drug area under the concentration–time curve to the minimum inhibitory concentration,^31^ so a higher dose could plausibly improve outcome. In our cohort, the observed mortality trend did not persist after adjustment, and CART found no mortality cutpoint. Mortality estimates therefore did not demonstrate an independent dose association, but were imprecise and cannot exclude clinically important benefit or harm. The unadjusted DOOR analysis found no statistically significant group difference; it did not establish equivalence. These findings support an exploratory renal safety signal in the upper recommended dose range rather than an optimal dose or confirmed clinical ceiling.

Inhaled colistin was more frequent in the nephrotoxicity group on univariable testing but did not persist on adjustment (aOR 1.42, 95% CI 0.69–2.94), as a time-varying exposure (time-dependent Cox 0.97, 95% CI 0.62–1.51) or in a Fine–Gray competing-risks model (sHR 0.94, 95% CI 0.62–1.44). The study was not designed to evaluate inhaled colistin and not all patients had pneumonia, so we draw no firm conclusion.

This study has limitations. It was single centre and retrospective, although the cohort was well characterized and the dose–nephrotoxicity association was consistent across methods. Isolates were not retained, so polymyxin B minimum inhibitory concentrations were not measured and the analysis is dose-based rather than exposure-based; colistin susceptibility was determined by an automated system rather than reference broth microdilution, though local polymyxin resistance is low,^32^ making misclassification unlikely to matter. Plasma polymyxin B concentrations were likewise not measured, so the weight-based dose, rather than individual exposure, was our metric; its correspondence with toxic exposure rests on population pharmacokinetics. Because interpatient variability persists despite weight-based dosing, prospective therapeutic drug-monitoring studies are needed to test the proposed risk region against measured exposure. As a single-centre study, generalizability is limited. The cohort was clinically heterogeneous, reflecting real-world use, and residual confounding remains possible despite adjustment. Finally, the sample size may have been underpowered for mortality; the observed renal association and proposed risk region require external validation, and the null DOOR comparison should not be interpreted as equivalence. Patients were classified by the initial maintenance dose; although this dose was unchanged in 92.6% of the nephrotoxicity-evaluable cohort and the association persisted when the analysis was restricted to those patients, dose reduction after renal injury would misclassify exposure and cannot be excluded. Disease severity was captured by the qSOFA and Pitt bacteraemia scores and by intensive care unit status rather than by a full severity index such as SOFA or APACHE II, which require serial physiological and laboratory data that were incompletely documented in this retrospective cohort; the dose estimate was stable across these adjustments (Table S3), but residual confounding by severity remains possible.

### Conclusion

Higher polymyxin B maintenance doses were associated with greater nephrotoxicity. Exploratory analyses suggested increased risk in the upper part of the recommended dosing range, above approximately 2.75 mg/kg/day. Mortality was not independently associated with dose, and the estimates did not exclude a clinically important effect at higher doses. As a single-centre retrospective analysis without therapeutic drug monitoring or external validation, this data-derived threshold should be regarded as hypothesis-generating rather than a clinically established dose ceiling.

## Supplementary Material

dkag322_Supplementary_Data

## Data Availability

The datasets generated during and/or analysed during the current study are available from the corresponding author on reasonable request.

## Supplementary data

Figures S1(a and b) and S2 and Tables S1–S6 are available as Supplementary data at *JAC* Online.
