# Supplementary material for: Weight-based polymyxin B dosing for mortality and nephrotoxicity: an exploratory dose–response study
Source: J Antimicrob Chemother. 2026 Sep 18;81(10):dkag322. doi: 10.1093/jac/dkag322 (PMC13586766; doi:10.1093/jac/dkag322)
Supplement: Supplementary file 1 [file dkag322_supplementary_data.docx]

**Supplementary data**

Weight-based polymyxin B dosing for mortality and nephrotoxicity: an exploratory dose-response study

Sung-Ching Pan^1,†^, Ming-Tao Tsai^2,†^, Jann-Tay Wang^1^, Wang-Huei Sheng^1^, Yu-Chung Chuang^1,*^

^1^Department of Internal Medicine, National Taiwan University Hospital, Taipei, Taiwan

^2^Department of Medicine, National Taiwan University Cancer Center, Taipei, Taiwan

^†^These authors contributed equally to this work.

* Corresponding author. Department of Internal Medicine, National Taiwan University Hospital, No. 7 Chung-Shan South Road, Taipei, Taiwan, 10002
E-mail address: weischuang@gmail.com (Y.-C. Chuang).

Telephone: +886-2-2312-3456. Ext 265054

**Table of Contents**

Supplementary Table S1……………………………………………………………….3

Supplementary Table S2……………………………………………….………………4

Supplementary Table S3……………………………………………….………………5

Supplementary Table S4……………………………………………….………………8

Supplementary Table S5……………………………………………….………………9

Supplementary Table S6……………………………………………….………………10

Supplementary Figure S1……………………………………………….……………...12

Supplementary Figure S2……………………………………………….………………13

Table S1. Cox proportional-hazards regression for 28-day mortality.

|  | **Model 1^a^** | | **Model 2^b^** | | **Model 3^c^** | |
| --- | --- | --- | --- | --- | --- | --- |
| **Predictor** | **aHR (95% CI)** | **p value** | **aHR (95% CI)** | **p value** | **aHR (95% CI)** | **p value** |
| Age, per year | 1.02 (1.01–1.04) | 0.01 | 1.02 (1.00–1.04) | 0.01 | 1.02 (1.00–1.04) | 0.01 |
| qSOFA, per point | 1.80 (1.33–2.45) | <0.001 | 1.82 (1.34–2.47) | <0.001 | 1.85 (1.36–2.51) | <0.001 |
| C-reactive protein, per mg/dL | 1.03 (1.01–1.06) | 0.02 | 1.04 (1.01–1.06) | 0.01 | 1.03 (1.01–1.06) | 0.01 |
| Serum albumin, per g/dL | 0.56 (0.33–0.95) | 0.03 | 0.60 (0.36–1.02) | 0.06 | 0.59 (0.35–1.00) | 0.05 |
| Platelet count, per 10⁴/µL | 0.95 (0.93–0.98) | <0.001 | 0.95 (0.93–0.98) | <0.001 | 0.95 (0.93–0.98) | <0.001 |
| Maintenance dose, per mg/kg/day | — | — | 0.50 (0.23–1.09) | 0.08 | — | — |
| Dose ≥ 2.5 mg/kg/day | — | — | — | — | 0.69 (0.40–1.21) | 0.19 |

*^a^* Using the covariates selected in the final logistic model (Table 2).

*^b^* With the continuous polymyxin B maintenance dose forced in.

^c^ With the dichotomized polymyxin B dose (≥ 2.5 mg/kg/day) forced in.

Abbreviations: qSOFA, quick Sequential Organ Failure Assessment; aHR, adjusted hazard ratio.

Table S2. Multiple imputation sensitivity analysis for the final 28-day mortality model.

| **Predictor** | **Complete case (n = 169)*ᵃ*** | **p value** | **Multiple imputation (n = 208)*ᵇ*** | **p value** |
| --- | --- | --- | --- | --- |
| Age, per year | 1.03 (1.01–1.06) | 0.02 | 1.02 (1.00–1.05) | 0.03 |
| qSOFA, per point | 1.76 (1.17–2.65) | 0.007 | 1.68 (1.19–2.38) | 0.004 |
| C-reactive protein, per mg/dL | 1.06 (1.01–1.11) | 0.02 | 1.05 (1.01–1.10) | 0.02 |
| Serum albumin, per g/dL | 0.51 (0.26–0.99) | 0.047 | 0.56 (0.31–1.03) | 0.06 |
| Platelet count, per 10⁴/µL | 0.95 (0.92–0.98) | 0.003 | 0.95 (0.93–0.98) | 0.002 |

Values are adjusted odds ratios (95% confidence interval).

*ᵃ* Complete-case analysis, as reported in Table 2 (169 of 208 patients, 81.2%).

*ᵇ* Multiple imputation by chained equations (20 imputations, predictive mean matching, fixed seed), using the full cohort of 208 patients. Missing data among the model covariates were confined to three baseline laboratory values: C-reactive protein in 33 patients (15.9%), serum albumin in 10 (4.8%) and platelet count in 4 (1.9%); all three were imputed. Age, qSOFA score, Charlson comorbidity index, maintenance dose and both outcomes were complete. Estimates from the two approaches are concordant.

Abbreviation: qSOFA, quick Sequential Organ Failure Assessment.

Table S3. Sensitivity of the dose–outcome associations to additional adjustment for disease severity.

| **Panel A. 28-day mortality** | | | | |
| --- | --- | --- | --- | --- |
| **Model** | **Dose ≥ 2.5 mg/kg/day, aOR (95% CI)** | **p value** | **Continuous dose, aOR (95% CI)** | **p value** |
| Primary model (Table 2)*ᵃ* | 0.55 (0.23–1.30) | 0.17 | 0.41 (0.12–1.37) | 0.15 |
| + Pitt bacteraemia score | 0.58 (0.24–1.38) | 0.22 | 0.41 (0.12–1.38) | 0.15 |
| + Mechanical ventilation | 0.54 (0.23–1.29) | 0.17 | 0.40 (0.12–1.35) | 0.14 |
| + Hypotension | 0.56 (0.24–1.33) | 0.19 | 0.43 (0.13–1.44) | 0.17 |
| + Intensive care unit at initiation | 0.54 (0.23–1.29) | 0.17 | 0.39 (0.12–1.33) | 0.13 |
| + Pitt bacteraemia score and intensive care unit | 0.58 (0.24–1.39) | 0.22 | 0.41 (0.12–1.39) | 0.15 |
| + Mechanical ventilation, hypotension and intensive care unit | 0.55 (0.23–1.32) | 0.18 | 0.40 (0.12–1.41) | 0.15 |
| **Panel B. Nephrotoxicity** | | | | |
| **Model** | **Dose ≥ 2.74 mg/kg/day, aOR (95% CI)** | **p value** | **Continuous dose, aOR (95% CI)** | **p value** |
| Primary model (Table 4)*ᵇ* | 2.60 (1.25–5.43) | 0.01 | 3.94 (1.17–13.27) | 0.03 |
| + Pitt bacteraemia score | 2.60 (1.25–5.43) | 0.01 | 3.93 (1.17–13.27) | 0.03 |
| + qSOFA score | 2.63 (1.26–5.49) | 0.01 | 4.01 (1.18–13.61) | 0.03 |
| + Mechanical ventilation | 2.60 (1.24–5.44) | 0.01 | 3.92 (1.16–13.23) | 0.03 |
| + Hypotension | 2.59 (1.24–5.41) | 0.01 | 3.91 (1.15–13.27) | 0.03 |
| + Intensive care unit at initiation | 2.59 (1.24–5.41) | 0.01 | 3.95 (1.17–13.35) | 0.03 |
| + Pitt bacteraemia score and intensive care unit | 2.58 (1.24–5.41) | 0.01 | 3.96 (1.17–13.37) | 0.03 |
| + Mechanical ventilation, hypotension and intensive care unit | 2.59 (1.23–5.43) | 0.01 | 3.98 (1.17–13.57) | 0.03 |

Each row is a separate model. Panel A models were fitted on the 169 patients with complete data for the final mortality model and retain its covariates (age, qSOFA, C-reactive protein, serum albumin and platelet count). Panel B models were fitted on all 163 nephrotoxicity-evaluable patients and retain the covariates of the final nephrotoxicity model (age, treatment duration and corticosteroid use). Continuous and dichotomized dose effects were estimated in separate models. The 2.5 mg/kg/day threshold used for mortality was pre-specified from the lower bound of the consensus dosing range, because recursive partitioning identified no mortality-specific cutpoint; the 2.74 mg/kg/day threshold used for nephrotoxicity was data-derived.

+, the listed variable was added to the covariates of the corresponding primary model.

*ᵃ* As reported in Table 2.

*ᵇ* As reported in Table 4.

Abbreviations: aOR, adjusted odds ratio; qSOFA, quick Sequential Organ Failure Assessment.

Table S4. Fine-Gray subdistribution-hazard model for nephrotoxicity, with death as a competing risk.

|  | **Continuous-dose model*^a,b^*** | | **Dichotomized ≥2.74 model*^a,b^*** | |
| --- | --- | --- | --- | --- |
| **Predictor** | **sHR (95% CI)** | **p value** | **sHR (95% CI)** | **p value** |
| Age, per year | 1.02 (1.01–1.03) | <0.001 | 1.02 (1.01–1.03) | <0.001 |
| Systemic corticosteroid use | 0.54 (0.35–0.84) | 0.006 | 0.56 (0.36–0.85) | 0.007 |
| Maintenance dose, per mg/kg/day | 2.01 (0.97–4.16) | 0.06 | — | — |
| Dose ≥ 2.74 mg/kg/day | — | — | 1.67 (1.04–2.67) | 0.03 |

***^a^*** Using the covariates selected in the final logistic model (Table 4), except treatment duration, which is represented by the time axis.

***^b^*** Two models differing only in dose parameterization: continuous maintenance dose versus dose dichotomized at ≥ 2.74 mg/kg/day.

Abbreviation: sHR, subdistribution hazard ratio.

Table S5. Ordinal proportional-odds regression of RIFLE class.

|  | **Continuous-dose model** ***^a,b^*** | | **Dichotomized ≥2.74 model *^a,b^*** | |
| --- | --- | --- | --- | --- |
| **Predictor** | **OR (95% CI)** | **p value** | **OR (95% CI)** | **p value** |
| Age, per year | 1.02 (1.00–1.04) | 0.03 | 1.02 (1.00–1.04) | 0.04 |
| Polymyxin B duration, per day | 1.13 (1.07–1.19) | <0.001 | 1.13 (1.07–1.19) | <0.001 |
| Systemic corticosteroid use | 0.72 (0.38–1.38) | 0.33 | 0.75 (0.39–1.44) | 0.39 |
| Maintenance dose, per mg/kg/day | 3.16 (1.14–8.76) | 0.03 | — | — |
| Dose ≥ 2.74 mg/kg/day | — | — | 2.12 (1.13–3.98) | 0.02 |

*^a^* Using the covariates selected in the final logistic model (Table 4).

*^b^* Two models: continuous maintenance dose versus dose dichotomized at ≥ 2.74 mg/kg/day; odds ratios are for a one-grade-worse RIFLE class.

Abbreviation: RIFLE, Risk, Injury, Failure, Loss, and End-stage kidney disease.

Table S6. Twenty-eight-day mortality and nephrotoxicity by maintenance-dose category.

| **Outcome** | **Categorization*^a^*** | **Maintenance dose, mg/kg/day** | **N** | **Events (%)** | **OR (95% CI)*^b^*** | **p*^c^*** | **Trend p*^d^*** |
| --- | --- | --- | --- | --- | --- | --- | --- |
| 28-day mortality | 3-category | <2.5 | 43 | 23 (53%) | 1 (reference) |  | 0.05 |
| 28-day mortality | 3-category | ≥2.5–<2.75 | 44 | 19 (43%) | 0.66 (0.28–1.54) | 0.34 | 0.05 |
| 28-day mortality | 3-category | ≥2.75 | 121 | 44 (36%) | 0.50 (0.25–1.01) | 0.05 | 0.05 |
| Nephrotoxicity | 3-category | <2.5 | 32 | 12 (38%) | 1 (reference) |  | 0.02 |
| Nephrotoxicity | 3-category | ≥2.5–<2.75 | 30 | 11 (37%) | 0.96 (0.34–2.71) | 0.95 | 0.02 |
| Nephrotoxicity | 3-category | ≥2.75 | 101 | 58 (57%) | 2.25 (0.99–5.09) | 0.05 | 0.02 |
| 28-day mortality | 4-category | <2.5 | 43 | 23 (53%) | 1 (reference) |  | 0.08 |
| 28-day mortality | 4-category | ≥2.5–<2.75 | 44 | 19 (43%) | 0.66 (0.28–1.54) | 0.34 | 0.08 |
| 28-day mortality | 4-category | ≥2.75–<3.0 | 95 | 34 (36%) | 0.48 (0.23–1.01) | 0.05 | 0.08 |
| 28-day mortality | 4-category | ≥3.0 | 26 | 10 (38%) | 0.54 (0.20–1.47) | 0.23 | 0.08 |
| Nephrotoxicity | 4-category | <2.5 | 32 | 12 (38%) | 1 (reference) |  | 0.005 |
| Nephrotoxicity | 4-category | ≥2.5–<2.75 | 30 | 11 (37%) | 0.96 (0.34–2.71) | 0.95 | 0.005 |
| Nephrotoxicity | 4-category | ≥2.75–<3.0 | 75 | 39 (52%) | 1.81 (0.77–4.21) | 0.17 | 0.005 |
| Nephrotoxicity | 4-category | ≥3.0 | 26 | 19 (73%) | 4.52 (1.47–13.92) | 0.008 | 0.005 |

*^a^* Pre-specified three- and four-level dose categories.

*^b^* Lowest category = reference.

^c^ p value for the odds ratio versus the reference category.

^d^ p value for the linear trend across the ordered dose categories.

Figure S1. Survival and cumulative incidence by polymyxin B maintenance-dose group. (A) Kaplan-Meier 28-day survival, with dose grouped at 2.5 mg/kg/day. (B) Cumulative incidence of nephrotoxicity, treating death as a competing risk, with dose grouped at 2.74 mg/kg/day. Numbers at risk are shown below each panel.

Figure S2. RIFLE class by polymyxin B maintenance-dose group (split at 2.74 mg/kg/day). Stacked bars show the percentage of patients in each class. A patient receiving ≥ 2.74 mg/kg/day had a worse RIFLE grade than a lower-dose patient 62.3% of the time (95% CI 54.1–70.1; Wilcoxon-Mann-Whitney *P* = 0.006).

Abbreviation: RIFLE, Risk, Injury, Failure, Loss, and End-stage kidney disease (ESKD).
